# MRI segmentation analysis in temporal lobe and idiopathic generalized epilepsy

**DOI:** 10.1186/1471-2377-14-131

**Published:** 2014-06-17

**Authors:** Hila Goldberg, Arie Weinstock, Niels Bergsland, Michael G Dwyer, Osman Farooq, Mona Sazgar, Guy Poloni, Cierra Treu, Bianca Weinstock-Guttman, Murali Ramanathan, Robert Zivadinov

**Affiliations:** 1 Comprehensive Epilepsy Program, State University of New York, Buffalo, NY, USA; 2 Bar-Ilan’s Faculty of Medicine in the Galilee, Safed, Israel; 3 Department of Neurology, State University of New York, Buffalo, NY, USA; 4 Buffalo Neuroimaging Analysis Center, The Jacobs Neurological Institute, State University of New York, Buffalo, NY, USA; 5 Department of Neurology, University of California, Orange, Irvine, CA, USA; 6 Department of Pharmaceutical Sciences, State University of New York, Buffalo, NY, USA; 7 Department of Neurology, Comprehensive Epilepsy Program, Women and Children’s Hospital of Buffalo, 219 Bryant Street, Buffalo, NY 14222, USA

**Keywords:** Temporal lobe epilepsy, Idiopathic generalized epilepsy, MRI segmentation, Brain atrophy

## Abstract

**Background:**

Temporal lobe epilepsy (TLE) and idiopathic generalized epilepsy (IGE) patients have each been associated with extensive brain atrophy findings, yet to date there are no reports of head to head comparison of both patient groups. Our aim was to assess and compare between tissue-specific and structural brain atrophy findings in TLE to IGE patients and to healthy controls (HC).

**Methods:**

TLE patients were classified in TLE lesional (L-TLE) or non-lesional (NL-TLE) based on presence or absence of MRI temporal structural abnormalities. High resolution 3 T MRI with automated segmentation by SIENAX and FIRST tools were performed in a group of patients with temporal lobe epilepsy (11 L-TLE and 15 NL-TLE) and in15 IGE as well as in 26 HC. Normal brain volume (NBV), normal grey matter volume (NGMV), normal white matter volume (NWMV), and volumes of subcortical deep grey matter structures were quantified. Using regression analyses, differences between the groups in both volume and left/right asymmetry were evaluated. Additionally, laterality of results was also evaluated to separately quantify ipsilateral and contralateral effects in the TLE group.

**Results:**

All epilepsy groups had significantly lower NBV and NWMV compared to HC (p < 0.001). L-TLE had lower hippocampal volume than HC and IGE (p = 0.001), and all epilepsy groups had significantly lower amygdala volume than HC (p < = 0.004). In L-TLE, there was evidence of atrophy in both ipsilateral and contralateral structures.

**Conclusions:**

Our study revealed that TLE and IGE patients demonstrated similar overall tissue-specific brain atrophy, although specific structures differences were appreciated. L-TLE also appeared to behave differently than NL-TLE, with atrophy not limited to the ipsilateral side.

## Background

Temporal lobe epilepsy (TLE) is the most common cause of partial epilepsy, and mesial temporal sclerosis (MTS) is the major pathological finding, occurring in roughly 50% of TLE patients. An estimated 30% of patients exhibit other identifiable magnetic resonance imaging (MRI) findings such as cortical dysplasia, low grade tumors or cavernous hemangiomas. The remaining 20% have no definite abnormalities observed visually on qualitative MRI assessment, and are often referred as non-lesional TLE [1] (NL TLE). Identifying the specific structures and neuronal pathways affected in TLE can help further understand the underlying mechanisms and disease chronicity. Different tissue-specific atrophy studies have been reported separately in epileptic syndromes including TLE, extra-temporal epilepsy, and idiopathic generalized epilepsy (IGE). In TLE, hippocampal involvement has been considerably investigated by various methods of MRI volumetric analyses, both manual and automatic [2-7]. Most studies have found significant reductions in hippocampal volumes, predominantly ipsilateral to the seizure focus [4-6], although relation to disease duration and seizure severity remains controversial [8-12]. Additional studies in TLE have reported more extensive structural involvement outside the temporal structures [9,10,13], in particular bilateral atrophy of the thalami has been consistently reported [9,11,14-16].

IGE are a group of age-related epilepsies with complex genetic backgrounds, subdivided according to the predominant seizure types (absence, myoclonic, or generalized tonic-clonic) and age of onset. The IGE are typically divided in the following sub-syndromes: childhood absence epilepsy (CAE), juvenile absence epilepsy (JAE), juvenile myoclonic epilepsy (JME), and IGE with generalized tonic-clonic seizures [17]. In IGE, various volumetric studies have reported findings of structural abnormalities [18-23], though reports implicating the thalamus are still somewhat contradictory [15,19-22,24]. While thalamic volumes in patients with IGE were not significantly different from those of normal control subjects in some reports [15], other studies reported evidence of regional atrophy in the thalamus, putamen and globus pallidus in IGE patients as compared to controls [20,22]. Although specific structural atrophies were reported independently in both TLE and IGE, there are no reports of head to head comparison of both patient groups using the same atrophy analysis measures.

The goal of this study was to assess the extent of tissue-specific and structural brain atrophy in patients with TLE compared with IGE and age-matched controls. We used an automated software tool for brain MRI segmentation into various regions of interest to enable quantitative analysis of the different brain structures [25,26].

## Methods

### Research design

This was a retrospective study conducted at the Buffalo Neuroimaging Analysis Center (BNAC) and the Comprehensive Epilepsy Program at the Jacobs Neurological Institute, Department of Neurology, State University of New York at Buffalo, with approval of the study protocol by the institutional review board (IRB). The study consisted of comprehensive review of medical records. Brain MRI segmentation analysis was performed on the previously performed MRI. A waiver of informed consent was obtained from the IRB.

### Study population

The study included three population groups: TLE patients, IGE patients and healthy controls. The first two patient population groups were retrieved through a patient epilepsy monitoring unit (EMU) database following IRB approval. All patient demographics were de-identified. The inclusion criteria for TLE patients consisted of: age >18 years at time of MRI, diagnosis of TLE supported by history, documented seizures on EMU long term monitoring (LTM) video electroencephalogram (EEG), and having underwent a 3 T MRI using a standard epilepsy protocol at a single site within 12 months of the LTM. The TLE patients’ were further subdivided into lesional (L-TLE) and non-lesional (NL-TLE) based on the presence or absence of temporal pathology on MRI as identified by the report of a certified neuro-radiologist. The inclusion criteria for IGE patients consisted of: age > 18 years, and supportive ictal findings on LTM. The IGE patients’ MRI were classified as normal or with a low number of non-specific white matter changes not related to the subcortical deep grey matter structures. The exclusion criteria included any MRI-detected structural abnormalities beyond abnormalities seen in TLE that would preclude the segmentation procedure. We enrolled only patients with TLE and IGE that were 18 years and older, as we only had age-matched MRI controls for this age group.

Clinical data of all TLE and IGE patients were obtained from medical history and LTM reports, and included location of epileptic focus (for TLE patients), International League Against Epilepsy seizure classification, frequency of seizures, age at epilepsy onset and duration of disease.

### MRI acquisition

All subjects underwent MRI testing at a single 3 T GE Signa Excite HD 12.0 Twin Speed 8-channel scanner (General Electric, Milwaukee, WI). Volumetric analysis was based on an axial T_1_ Inversion Recovery Fast Spoiled Gradient Echo (IR-FSPGR) sequence with flip angle = 20°, repetition time = 9.46 ms, echo time = 3.87 ms, matrix size of 256×256 pixels, and voxels of 1 × 1 × 1.5 mm. The lesions were assessed on 2D scans (proton density [PD]/T2, Fluid attenuated inversion recovery [FLAIR] and spin echo [SE] T1), with 48 slices collected, with a thickness of 3 mm, and no gap between slices.

### Image processing and volumetric analysis

To all images we applied an automatic inhomogeneity correction [27] to overcome distortions of intensity non-uniformity created by the scanner. The volumetric analyses were performed with the use of FMRIB tools (Oxford Centre for Functional MRI of the Brain, version 4.1) [25,26]. The volumetric analysis was performed in a blinded manner in regard to the qualitative MRI results provided by the neuro-radiologist.

**Figure 1:**
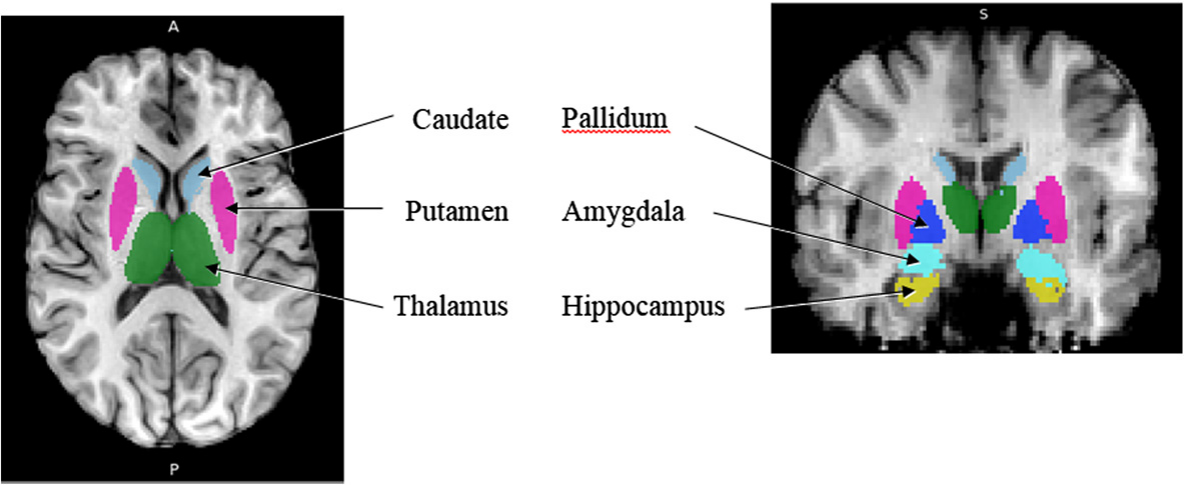
An axial and coronal MRI slice demonstrating the FIRST segmentation of the subcortical deep GM structures.

The first stage of analysis used the structural image evaluation using normalization of atrophy, cross-sectional (SIENAX) [28,29] to estimate the normalized brain volume (NBV), normalized grey matter volume (NGMV), and normalized white matter volume (NWMV). SIENAX starts by extracting brain and skull images from single whole-head input data [30]. The brain image is then affine-registered to MNI152 space [31,32], using the skull image to determine the registration scaling factor (to be used as a normalization for head size). Next, tissue-type segmentation with partial volume estimation is carried out in order to calculate the total volume of brain tissue (including separate estimates of grey and white matter volumes) [33]. The second stage of analysis used FIRST (FMRIB’s Integrated Registration and Segmentation Tool) [34-36] to estimate the volumes of the following subcortical deep grey matter structures in both hemispheres: hippocampus, amygdala, thalamus, putamen, pallidum and caudate. FIRST is a model-based automated segmentation/registration tool. The shape models used in FIRST are constructed from manually segmented images provided by the Center for Morphometric Analysis, Massachusetts General Hospital, Boston. The manual labels are parameterized as surface meshes and modeled as a point distribution model. Deformable surfaces are used to automatically parameterize the volumetric labels in terms of meshes. The deformable surfaces are constrained to preserve vertex correspondence across the training data. Furthermore, normalized intensities along the surface normals are sampled and modeled. The shape and appearance model is based on multivariate Gaussian assumptions. Shape is then expressed as a mean with modes of variation (principal components). Based on learned models, FIRST searches through linear combinations of shape modes of variation for the most probable shape instance given the observed intensities in the T1 image. An example of a segmented brain is presented in Figure 1.

### Statistical analysis

Statistical analysis was performed with R version 3.1.0 (http://www.R-project.org/). A GLM-based analysis of covariance (ANCOVA) model was used to evaluate group differences in volume measures between controls, IGE, NL-TLE, and L-TLE while controlling for variation in age and gender. Where group was a significant factor, post-hoc pair-wise comparisons were performed to identify specific differences. In the primary set of analyses, total tissue volumes and bilateral structure volumes were compared. In a secondary set of analyses, laterality was evaluated by comparing left/right asymmetry between groups. Asymmetry was calculated as the absolute difference between left and right structures divided by the total volume (left + right). Finally, ipsilateral and contralateral structures (as related to epileptic focus localization) in L-TLE were compared to HC to evaluate whether there was evidence of contralateral atrophy. For this final analyses, individual rather than left/right averaged structure volumes were used, so a mixed-effect model was employed using laterality, age, and gender as fixed effects and subject as a random effect. We used a conservative type 1 error threshold of p < 0.01 to correct for multiple testing.

## Results

### Demographic and clinical characteristics of the study groups

Demographic and clinical information of patients and controls is presented in Table 1. The epilepsy populations were initially composed of 44 patients diagnosed with TLE and 30 patients with IGE. Eighteen TLE and 15 IGE subjects were consequently dropped due to exclusion criteria of having no recorded ictal events during LTM, having multifocal seizure onset (only for TLE), or other MRI-detected abnormalities (brain tumor, multiple sclerosis, sub-optimal MRI study, etc.) that would affect the segmentation procedure. The final study groups consisted of 26 patients with unilateral TLE (15 NL-TLE and 11 L-TLE), 15 patients with IGE and 26 healthy controls. There were no significant differences between the groups’ demographic distributions, other than a female predisposition for IGE patients as compared to TLE and controls. IGE patients were also of notably younger ages.

**Table 1:** Demographic and clinical characteristics

**Characteristic**	**TLE**	**IGE**	**HC**
N (Males: Females)	26 (14:12)	15 (4:11)	26 (11:15)
Age, years	42.1 ± 17.2 (18–72)	31.7 ± 11.7 (18–59)	38.6 ± 14.3 (19–61)
Age of onset, years	24.1 ± 20.3	12.5 ± 6.5	–
Epilepsy duration in years	17.9 ± 18.6	19.2 ± 15.6	–
Seizure frequency per month*	2.0	0.2	–
MRI findings	15 non-lesional, 11 lesional	All non-lesional	All non-lesional

*Seizure frequency is median (25^th^-75^th^ inter-quartile range).

Legend: *TLE* temporal lobe epilepsy; *IGE* idiopathic generalized epilepsy; *HC* healthy controls; *SD* standard deviation.

Continuous variables are shown as mean ± SD.

In the TLE group, 16 had the seizure focus on the left hemisphere and 10 in the right hemisphere (for L-TLE alone, 4 right, 7 left). Fifteen patients in the TLE group had complex partial seizures with secondary generalization where 11 had complex partial seizures without secondary generalization. In the IGE group, 14/15 subjects had generalized tonic-clonic seizures, 11/15 had absence seizure and myoclonic seizures. MRI abnormalities included hippocampal atrophy in 5 patients and other findings in the 6 patients (cortical dysplasia 1, venous anomaly 1, atrophy 2, and non-specifc juxtacortical lesions 2).

### Tissue- and structure-specific atrophy comparisons

**Figure 2:**
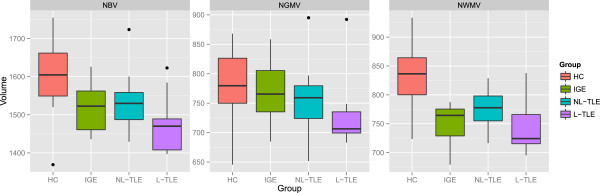
**Boxplots showing normalized brain parenchymal volume (NBV), normalized gray matter volume (NGMV), and normalized white matter volume (NWMV) in patients with IGE, NL-TLE, L-TLE, and healthy controls (HC).** Volumes are in cm^3^.

**Figure 3:**
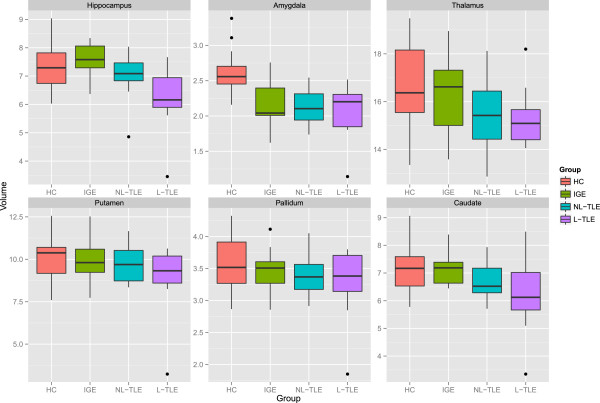
**Volumes of the amygdala, caudate, hippocampus, thalamus, pallidum and putamen in patients with IGE, L-TLE, NL-TLE, and in healthy controls (HC).** Volumes are in cm^3^. Standard error bars are presented.

**Figure 4:**
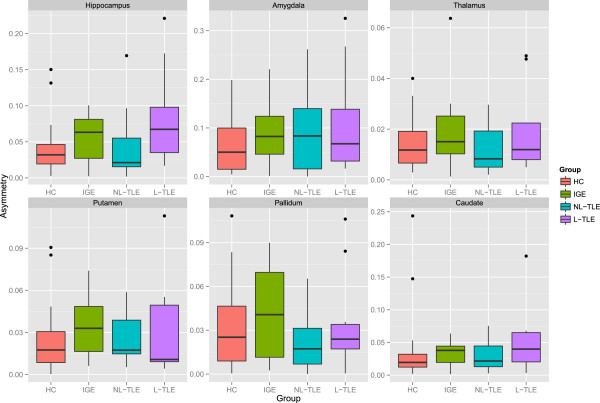
**Left/right asymmetry in volumes of the amygdala, caudate, hippocampus, thalamus, pallidum and putamen in patients with IGE, L-TLE, NL-TLE, and in healthy controls (HC).** Volumes are in cm^3^.

**Figure 5:**
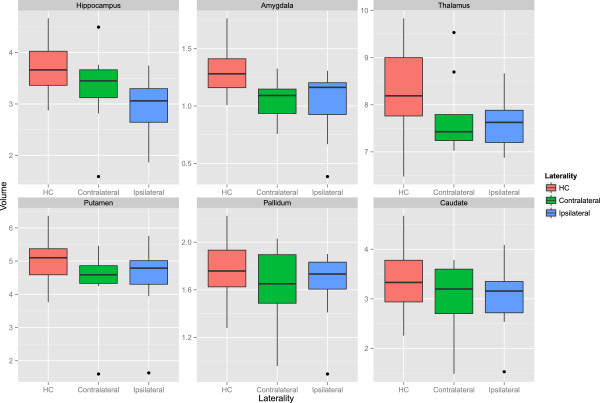
**Ipsilaterl and contralateral volumes of the amygdala, caudate, hippocampus, thalamus, pallidum and putamen in patients L-TLE and in healthy controls (HC).** Volumes are in cm^3^.

Figure 2 compares the tissue-specific volumetric measures between groups. After correcting for age and gender, NBV (F = 13.72, *p* < 0.001) and NWMV (F = 16.32, p < 0.001) were significantly different between groups. Post-hoc analysis showed that HC had greater NBV and NWMV as compared to all epilepsy groups (p < 0.001). This indicates that the whole brain volume changes in epilepsy are predominantly the result of WM volume loss. Within epilepsy groups, there were no significant tissue-wide differences, although there was a general trend for L-TLE to have the lowest volumes.Figure 3 compares structure-specific volumetric measures between groups. There were significant group effects in the hippocampus (F = 7.18, p = 0.001), amygdala (F = 14.77, p < 0.001), and caudate (F = 4.56, p = 0.006), and a trend in the thalamus (F = 3.95, p = 0.012). Post-hoc analysis between groups in the significant structures revealed lower hippocampal volume in L-TLE compared to both HC (p = 0.001) and IGE (p < 0.001), lower amygdala volume in all epilepsy groups compared to HC (p < = 0.004), and a trend for lower caudate volume in L-TLE compared to HC (p = 0.012) and IGE (p = 0.042).Figure 4 compares asymmetry between structures and groups. There were no statistically significant differences between groups for any structures, although there was a weak trend for L-TLE to have more hippocampal asymmetry than HC (p = 0.071).Figure 5 shows the results of laterality analysis between the L-TLE and HC groups. For the hippocampus, the ipsilateral side was significantly smaller than HC (p < 0.001), with a trend for the contralateral side as well (p = 0.03). Both ipsilateral and contralateral amygdalae were significantly smaller than in HC (p < 0.001). Putamen differences were not significant, but showed trends for both ipsilateral (p = 0.044) and contralateral (p = 0.0101). There were similar bilateral non-significant trends in ipsilateral (p = 0.02) and contralateral (p = 0.02) pallidum. No significant differences were observed in the thalamus or caudate. Within the L-TLE group, only the hippocampus showed a trend toward lower volume in the ipsilateral vs. contralateral side (p = 0.011).

## Discussion

In this study we compared L-TLE, NL-TLE, IGE, and healthy controls using the same methodology and same 3 T-scanner. Our study revealed that patients with TLE and IGE demonstrated similar tissue-specific atrophies in the whole brain and white matter. After correcting for age and gender, normal brain volume, normal grey matter volume and normal white matter volume were lower in the epilepsy group (TLE plus IGE) compared to controls, but predominantly as a result of white matter volume loss.

Our results in L-TLE patients were similar to varying TLE study reports in relation to atrophy at various subcortical structures such as the hippocampus and basal ganglia [6,9,11,13,15,37]. The extent of atrophy noted in TLE patients suggests that the impact of temporal seizures is more widespread than the immediate temporal vicinity of the epileptogenic region. Furthermore, the bilateral distribution of tissue-specific atrophy suggests that the neuronal atrophy extends to both hemispheres, regardless of the side of focal epileptic origin [38-40].

Our results suggest that patients with chronic epilepsy, whether TLE or IGE, have chronic atrophy, mostly of white matter and of various subcortical deep grey matter structures: particularly hippocampi and amygdale bilaterally. Altered white matter integrity has been reported in TLE, with association to cognitive and clinical profiles as measured on diffusion tensor imaging (DTI) studies in the temporal, cerebellar and fronto-parietal structures [41-43]. Extensive white matter tracts abnormalities on DTI were identified also in JME [44].

Findings of ipsilateral thalamic hypometabolism on positron emission tomography (PET) studies have been described in patients with TLE, often attributed to a diaschisis effect. It has been postulated that hippocampal cell loss may result in decreased efferent synaptic activity to the thalamus and basal ganglia, causing decreased neuronal activity in these structures with consequent hypometabolism. It remains unknown whether the process of subcortical deep grey matter atrophy seen in volumetric studies is due to a similar mechanism to the ipsilateral hypometabolism seen in PET studies in TLE patients [45,46].

Several limitations in our study which may have impacted our results and statistical power should be acknowledged. Our study was retrospective, and included a relatively small patient sample. Consesquently this might have altered our ability to detect subtle volume changes. In particular, we saw many intriguing statistical trends that should be investigated in a larger study. In addition, we performed a cross-sectional evaluation, making it difficult to ascertain progressive developments. We also did not have sufficient power to analyze the impact of medication, which may have modified atrophy rates. Another limitation may be that the IGE group was younger and although we corrected for age in our analysis the earlier onset age of epilepsy in this group may be an interfering factor.

## Conclusion

In conclusion, our study supports that TLE and IGE are both associated with significant atrophy compared to healthy controls These changes appear to occur beyond the local temporal epileptogenic region for TLE patients. It remains unknown whether these changes are associated with neurological and cognitive morbidities often seen in patients with chronic epilepsy.

### Ethical approval

Prior to the initiation of the study, approval was obtained from the Institutional Review Board of the State University of New York at Buffalo.

## Abbreviations

TLE: Temporal lobe epilepsy; IGE: Idiopathic generalized epilepsy; MRI: Magnetic resonance imaging; NBV: Normal brain volume; NGMV: Normal gray matter volume; NWMV: Normal white matter volume; MTS: Mesial temporal sclerosis; LTM: Long term monitoring; AED: Anti-epileptic drug; CAE: Childhood absence epilepsy; JAE: Juvenile absence epilepsy; JME: Juvenile myoclonic epilepsy; EMU: Epilepsy monitoring unit; LTM: Long term monitoring; EEG: Electroencephalogram.

## Competing interests

Arie Weinstock is part of the speaker bureau for Cyberonics and Supernus. He is the site Principal Investigator for multi-center studies sponsored by UCB pharma and Eisai.

Mona Sazgar is on the speaker’s bureau of UCB Pharma and have a grant with Lunbceck for an investigator initiated trial.

Murali Ramanathan serves as an editor for the American Association of Pharmaceutical Scientists Journal; receives royalties for publishing *The Pharmacy Calculations Workbook* (Pinnacle, Summit and Zenith, 2008): and has received research support from EMD Serono, Novartis, Pfizer, Monsanto, Department of Defense, the National Multiple Sclerosis Society, and the National Science Foundation. He has served as a consultant for Biogen Idec, Allegran and Netezza.

Bianca Weinstock-Guttman has participated in speaker’s bureaus and served as a consultant for Biogen Idec, Teva Neurosciences, EMD Serono, Pfizer, Novartis, Genzyme & Sanofi, Mylan and Acorda. She also has received grant/research support from the agencies listed above as well as Questcor and Shire. No other industry financial relationships exist.

Robert Zivadinov received personal compensation from Teva Pharmacuticals, Biogen Idec, EMD Serono, Novartis and Sanofi-Genzyme for speaking and consultant fees. Dr. Zivadinov received financial support for research activities from Biogen Idec, Teva Pharmacuticals, EMD Serono, Novartis and Sanofi-Genzyme.

Michael Dwyer has received consulting fees from EMD Serono and Claret Medical.

Hila Goldberg, Niels Bergsland, Osman Farooq, Guy Poloni, and Cierra Treu declare that they have no competing interests.

## Authors’ contributions

HG contributed to the study design and conduct, subject recruitment, data analysis and interpretation, critical review and drafting of the manuscript. AW contributed to the study conduct, subject recruitment, data analysis and interpretation, critical review, and drafting of the manuscript. NB contributed to data analysis, critical review and approval of the manuscript. MGD contributed to data analysis, statistical analysis, critical review and revision, and approval of the manuscript. OF contributed to data analysis, critical review and drafting of the manuscript. MS contributed to data analysis and interpretation, critical review and approval of the manuscript. GP contributed to data analysis, critical review and approval of the manuscript. CT contributed to data analysis, critical review and approval of the manuscript. BWG contributed to the study conduct, data analysis and interpretation, critical review, and drafting of the manuscript. MR performed statistical analysis and contributed to critical review and approval of the manuscript. RZ contributed to the study design and conduct, subject recruitment, data analysis and interpretation, critical review, and critical review and drafting of the manuscript. All authors read and approved the final manuscript.

## Pre-publication history

The pre-publication history for this paper can be accessed here:

http://www.biomedcentral.com/1471-2377/14/131/prepub

## References

[B1] Engel J Jr Introduction to temporal lobe epilepsy Epilepsy Res 1996 26 141 150 8985696 10.1016/s0920-1211(96)00043-5

[B2] Bonilha L Halford JJ Rorden C Roberts DR Rumboldt Z Eckert MA Automated MRI analysis for identification of hippocampal atrophy in temporal lobe epilepsy Epilepsia 2009 50 228 233 18727679 10.1111/j.1528-1167.2008.01768.x

[B3] Bonilha L Kobayashi E Rorden C Cendes F Li LM Medial temporal lobe atrophy in patients with refractory temporal lobe epilepsy J Neurol Neurosurg Psychiatry 2003 74 1627 1630 14638879 10.1136/jnnp.74.12.1627 PMC1757422

[B4] Garcia-Finana M Denby CE Keller SS Wieshmann UC Roberst N Degree of hippocampal atrophy is related to side of seizure onset in temporal lobe epilepsy AJNR Am J Neuroradiol 2006 27 1046 1052 16687541 PMC7975739

[B5] Hogan RE Wang L Bertrand ME Willmore LJ Bucholz RD Nassif AS Csernansky JG MRI-based high-dimensional hippocampal mapping in mesial temporal lobe epilepsy Brain 2004 127 1731 1740 15231583 10.1093/brain/awh197

[B6] McDonald CR Hagler DJ Jr Ahmadi ME Tecoma E Iragui V Dale AM Halgren E Subcortical and cerebellar atrophy in mesial temporal lobe epilepsy revealed by automatic segmentation Epilepsy Res 2008 79 130 138 18359198 10.1016/j.eplepsyres.2008.01.006 PMC2412955

[B7] Mueller SG Laxer KD Barakos J Cheong I Garcia P Weiner MW Subfield atrophy pattern in temporal lobe epilepsy with and without mesial sclerosis detected by high-resolution MRI at 4 Tesla: preliminary results Epilepsia 2009 50 1474 1483 19400880 10.1111/j.1528-1167.2009.02010.x PMC2804395

[B8] Bernasconi N Natsume J Bernasconi A Progression in temporal lobe epilepsy: differential atrophy in mesial temporal structures Neurology 2005 65 223 228 16043790 10.1212/01.wnl.0000169066.46912.fa

[B9] Dreifuss S Vingerhoets FJ Lazeyras F Andino SG Spinelli L Delavelle J Seeck M Volumetric measurements of subcortical nuclei in patients with temporal lobe epilepsy Neurology 2001 57 1636 1641 11706104 10.1212/wnl.57.9.1636

[B10] Seidenberg M Kelly KG Parrish J Geary E Dow C Rutecki P Hermann B Ipsilateral and contralateral MRI volumetric abnormalities in chronic unilateral temporal lobe epilepsy and their clinical correlates Epilepsia 2005 46 420 430 15730540 10.1111/j.0013-9580.2005.27004.x

[B11] Szabo CA Lancaster JL Lee S Xiong JH Cook C Mayes BN Fox PT MR imaging volumetry of subcortical structures and cerebellar hemispheres in temporal lobe epilepsy AJNR Am J Neuroradiol 2006 27 2155 2160 17110687 PMC7977233

[B12] Theodore WH Bhatia S Hatta J Fazilat S DeCarli C Bookheimer SY Gaillard WD Hippocampal atrophy, epilepsy duration, and febrile seizures in patients with partial seizures Neurology 1999 52 132 136 9921860 10.1212/wnl.52.1.132

[B13] Riederer F Lanzenberger R Kaya M Prayer D Serles W Baumgartner C Network atrophy in temporal lobe epilepsy: a voxel-based morphometry study Neurology 2008 71 419 425 18678824 10.1212/01.wnl.0000324264.96100.e0

[B14] Gong G Concha L Beaulieu C Gross DW Thalamic diffusion and volumetry in temporal lobe epilepsy with and without mesial temporal sclerosis Epilepsy Res 2008 80 184 193 18490143 10.1016/j.eplepsyres.2008.04.002

[B15] Natsume J Bernasconi N Andermann F Bernasconi A MRI volumetry of the thalamus in temporal, extratemporal, and idiopathic generalized epilepsy Neurology 2003 60 1296 1300 12707432 10.1212/01.wnl.0000058764.34968.c2

[B16] Labate A Cerasa A Gambardella A Aguglia U Quattrone A Hippocampal and thalamic atrophy in mild temporal lobe epilepsy: a VBM study Neurology 2008 71 1094 1101 18824674 10.1212/01.wnl.0000326898.05099.04

[B17] Jallon P Latour P Epidemiology of idiopathic generalized epilepsies Epilepsia 2005 46 Suppl 9 10 14 16302871 10.1111/j.1528-1167.2005.00309.x

[B18] Betting LE Mory SB Lopes-Cendes I Li LM Guerreiro MM Guerreiro CA Cendes F MRI reveals structural abnormalities in patients with idiopathic generalized epilepsy Neurology 2006 67 848 852 16966549 10.1212/01.wnl.0000233886.55203.bd

[B19] Ciumas C Savic I Structural changes in patients with primary generalized tonic and clonic seizures Neurology 2006 67 683 686 16924024 10.1212/01.wnl.0000230171.23913.cf

[B20] Du H Zhang Y Xie B Wu N Wu G Wang J Jiang T Feng H Regional atrophy of the basal ganglia and thalamus in idiopathic generalized epilepsy J Magn Reson Imaging 2011 33 817 821 21448945 10.1002/jmri.22416

[B21] Kim JH Lee JK Koh SB Lee SA Lee JM Kim SI Kang JK Regional grey matter abnormalities in juvenile myoclonic epilepsy: a voxel-based morphometry study Neuroimage 2007 37 1132 1137 17689105 10.1016/j.neuroimage.2007.06.025

[B22] Seeck M Dreifuss S Lantz G Jallon P Foletti G Despland PA Delavelle J Lazeyras F Subcortical nuclei volumetry in idiopathic generalized epilepsy Epilepsia 2005 46 1642 1645 16190937 10.1111/j.1528-1167.2005.00259.x

[B23] Woermann FG Sisodiya SM Free SL Duncan JS Quantitative MRI in patients with idiopathic generalized epilepsy. Evidence of widespread cerebral structural changes Brain 1998 121 1661 1667 9762955 10.1093/brain/121.9.1661

[B24] Bernhardt BC Rozen DA Worsley KJ Evans AC Bernasconi N Bernasconi A Thalamo-cortical network pathology in idiopathic generalized epilepsy: insights from MRI-based morphometric correlation analysis Neuroimage 2009 46 373 381 19385011 10.1016/j.neuroimage.2009.01.055

[B25] Smith SM Jenkinson M Woolrich MW Beckmann CF Behrens TE Johansen-Berg H Bannister PR De Luca M Drobnjak I Flitney DE Niazy RK Saunders J Vickers J Zhang Y De Stefano N Brady JM Matthews PM Advances in functional and structural MR image analysis and implementation as FSL Neuroimage 2004 23 Suppl 1 S208 219 15501092 10.1016/j.neuroimage.2004.07.051

[B26] Woolrich MW Jbabdi S Patenaude B Chappell M Makni S Behrens T Beckmann C Jenkinson M Smith SM Bayesian analysis of neuroimaging data in FSL Neuroimage 2009 45 S173 186 19059349 10.1016/j.neuroimage.2008.10.055

[B27] Sled JG Zijdenbos AP Evans AC A nonparametric method for automatic correction of intensity nonuniformity in MRI data IEEE Trans Med Imaging 1998 17 87 97 9617910 10.1109/42.668698

[B28] Smith SM De Stefano N Jenkinson M Matthews PM Normalized accurate measurement of longitudinal brain change J Comput Assist Tomogr 2001 25 466 475 11351200 10.1097/00004728-200105000-00022

[B29] Smith SM Zhang Y Jenkinson M Chen J Matthews PM Federico A De Stefano N Accurate, robust, and automated longitudinal and cross-sectional brain change analysis Neuroimage 2002 17 479 489 12482100 10.1006/nimg.2002.1040

[B30] Smith SM Fast robust automated brain extraction Hum Brain Mapp 2002 17 143 155 12391568 10.1002/hbm.10062 PMC6871816

[B31] Jenkinson M Bannister P Brady M Smith S Improved optimization for the robust and accurate linear registration and motion correction of brain images Neuroimage 2002 17 825 841 12377157 10.1016/s1053-8119(02)91132-8

[B32] Jenkinson M Smith S A global optimisation method for robust affine registration of brain images Med Image Anal 2001 5 143 156 11516708 10.1016/s1361-8415(01)00036-6

[B33] Zhang Y Brady M Smith S Segmentation of brain MR images through a hidden Markov random field model and the expectation-maximization algorithm IEEE Trans Med Imaging 2001 20 45 57 11293691 10.1109/42.906424

[B34] Patenaude B Smith SM Kennedy DN Jenkinson M A Bayesian model of shape and appearance for subcortical brain segmentation Neuroimage 2011 56 907 922 21352927 10.1016/j.neuroimage.2011.02.046 PMC3417233

[B35] Zivadinov R Heininen-Brown M Schirda CV Poloni GU Bergsland N Magnano CR Durfee J Kennedy C Carl E Hagemeier J Benedict RH Weinstock-Guttman B Dwyer MG Abnormal subcortical deep-gray matter susceptibility-weighted imaging filtered phase measurements in patients with multiple sclerosis: a case–control study Neuroimage 2012 59 331 339 21820063 10.1016/j.neuroimage.2011.07.045

[B36] Batista S Zivadinov R Hoogs M Bergsland N Heininen-Brown M Dwyer MG Weinstock-Guttman B Benedict RH Basal ganglia, thalamus and neocortical atrophy predicting slowed cognitive processing in multiple sclerosis J Neurol 2012 259 139 146 21720932 10.1007/s00415-011-6147-1

[B37] Pitkanen A Tuunanen J Kalviainen R Partanen K Salmenpera T Amygdala damage in experimental and human temporal lobe epilepsy Epilepsy Res 1998 32 233 253 9761324 10.1016/s0920-1211(98)00055-2

[B38] Diniz PR Velasco TR Salmon CE Sakamoto AC Leite JP Santos AC Extratemporal damage in temporal lobe epilepsy: magnetization transfer adds information to volumetric MR imaging AJNR Am J Neuroradiol 2011 32 1857 1861 21885719 10.3174/ajnr.A2639 PMC7965985

[B39] Araujo D Santos AC Velasco TR Wichert-Ana L Terra-Bustamante VC Alexandre V Jr Carlotti CG Jr Assirati JA Jr Machado HR Walz R Leite JP Sakamoto AC Volumetric evidence of bilateral damage in unilateral mesial temporal lobe epilepsy Epilepsia 2006 47 1354 1359 16922881 10.1111/j.1528-1167.2006.00605.x

[B40] Concha L Beaulieu C Gross DW Bilateral limbic diffusion abnormalities in unilateral temporal lobe epilepsy Ann Neurol 2005 57 188 196 15562425 10.1002/ana.20334

[B41] Concha L Beaulieu C Collins DL Gross DW White-matter diffusion abnormalities in temporal-lobe epilepsy with and without mesial temporal sclerosis J Neurol Neurosurg Psychiatry 2009 80 312 319 18977826 10.1136/jnnp.2007.139287

[B42] Gross DW Concha L Beaulieu C Extratemporal white matter abnormalities in mesial temporal lobe epilepsy demonstrated with diffusion tensor imaging Epilepsia 2006 47 1360 1363 16922882 10.1111/j.1528-1167.2006.00603.x

[B43] Riley JD Franklin DL Choi V Kim RC Binder DK Cramer SC Lin JJ Altered white matter integrity in temporal lobe epilepsy: association with cognitive and clinical profiles Epilepsia 2010 51 536 545 20132296 10.1111/j.1528-1167.2009.02508.x PMC2929974

[B44] Liu M Concha M Beaulieu C Gross DW Distinct white matter abnormalities in different idiopathic generalized epilepsy syndromes Epilepsia 2011 52 12 2267 2275 22092238 10.1111/j.1528-1167.2011.03313.x

[B45] Chang CP Yen DJ Yu SM Liu RS Chang HF Hsieh HJ Shih YH Chu LS Wang SJ Unilateral thalamic hypometabolism in patients with temporal lobe epilepsy J Formos Med Assoc 2008 107 567 571 18632416 10.1016/S0929-6646(08)60170-9

[B46] Dlugos DJ Jaggi J O'Connor WM Ding XS Reivich M O’Connor MJ Sperling MR Hippocampal cell density and subcortical metabolism in temporal lobe epilepsy Epilepsia 1999 40 408 413 10219265 10.1111/j.1528-1157.1999.tb00734.x

